# Validation of *N*-myristoyltransferase as Potential Chemotherapeutic Target in Mammal-Dwelling Stages of *Trypanosoma cruzi*

**DOI:** 10.1371/journal.pntd.0004540

**Published:** 2016-04-29

**Authors:** Linda J. Herrera, Stephen Brand, Andres Santos, Lilian L. Nohara, Justin Harrison, Neil R. Norcross, Stephen Thompson, Victoria Smith, Carolina Lema, Armando Varela-Ramirez, Ian H. Gilbert, Igor C. Almeida, Rosa A. Maldonado

**Affiliations:** 1 The Border Biomedical Research Center, Department of Biological Sciences, University of Texas at El Paso, El Paso, Texas, United States of America; 2 Drug Discovery Unit, Division of Biological Chemistry and Drug Discovery, College of Life Sciences, University of Dundee, Sir James Black Centre, Dundee, United Kingdom; Hospital Infantil de Mexico Federico Gomez, UNITED STATES

## Abstract

**Background:**

*Trypanosoma cruzi* causes Chagas disease, an endemic and debilitating illness in Latin America. Lately, owing to extensive population movements, this neglected tropical disease has become a global health concern. The two clinically available drugs for the chemotherapy of Chagas disease have rather high toxicity and limited efficacy in the chronic phase of the disease, and may induce parasite resistance. The development of new anti*-T*. *cruzi* agents is therefore imperative. The enzyme *N-*myristoyltransferase (NMT) has recently been biochemically characterized, shown to be essential in *Leishmania major*, *Trypanosoma brucei*, *and T*. *cruzi¸* and proposed as promising chemotherapeutic target in these trypanosomatids.

**Methodology/Principal Findings:**

Here, using high-content imaging we assayed eight known trypanosomatid NMT inhibitors, against mammal-dwelling intracellular amastigote and trypomastigote stages and demonstrated that three of them (compounds **1**, **5**, and **8**) have potent anti-proliferative effect at submicromolar concentrations against *T*. *cruzi*, with very low toxicity against human epithelial cells. Moreover, metabolic labeling using myristic acid, azide showed a considerable decrease in the myristoylation of proteins in parasites treated with NMT inhibitors, providing evidence of the on-target activity of the inhibitors.

**Conclusions/Significance:**

Taken together, our data point out to the potential use of NMT inhibitors as anti-*T*. *cruzi* chemotherapy.

## Author Summary

*Trypanosoma cruzi* is the etiological agent of Chagas disease, a chronic illness endemic to Latin America. In recent years, this neglected infectious disease has also become a global health concern due to the intense migratory flow of chronically affected individuals to nonendemic regions such as the U.S. and Europe. The only available drugs against this disease have important disadvantages, such as, high toxicity, strain resistance, and variable efficacy, underscoring the urgent need for new anti-*T*. *cruzi* agents. *N*-myristoyltransferase (NMT) catalyzes the attachment of myristic acid, a fatty acid, to many proteins. In *T*. *cruzi*, this enzyme appears to be essential, highlighting its potential as a drug target. In this study, we show that NMT is expressed in all stages of the parasite and localized to the endoplasmic reticulum. We also evaluate eight NMT inhibitors as anti-*T*. *cruzi* agents. We demonstrate the efficacy of three of these compounds as anti-proliferative agents in this intracellular parasite, while showing very low toxicity against mammalian cells. In addition, we provide evidence of the on-target effect of these compounds, thus demonstrating their specificity and validating NMT as a drug target. These inhibitors hold great potential for further exploration as anti-Chagas disease agents.

## Introduction

The flagellate protozoan parasite, *Trypanosoma cruzi*, is the etiologic agent of Chagas disease (or American trypanosomiasis), a neglected tropical disease widespread in Latin America. According to the World Health Organization (http://www.who.int/mediacentre/factsheets/fs340/en/), currently there are 6–7 million individuals chronically infected, resulting in over 15,000 deaths annually [1]. In recent years, Chagas disease has become an emerging public health concern in the U.S. and other nonendemic countries such as Spain, due to the immigration of asymptomatic infected individuals from endemic areas [2]. The current treatment for *T*. *cruzi* consists of two nitroheterocyclic derivatives, benznidazole and nifurtimox, which are very effective in the acute stage of the disease, but have limited efficacy in the chronic stage of the disease. Moreover, these compounds may cause severe side effects and induce parasite strain resistance. Therefore, there is an urgent need for new, more effective drugs to treat Chagas disease [3–5].

Myristoyl-CoA:protein *N*-myristoyltransferase (NMT) catalyzes the attachment of the fatty acid myristic acid (C14:0) to the amino-terminal glycine residue of many eukaryotic proteins [6]. NMT binds first to myristoyl-CoA, which results in a conformational change that allows the target (poly)peptide to bind next. The myristoyl-CoA:NMT-(poly)peptide complex leads to catalysis and the product, a *N*-myristoylated (poly)peptide, is released [7]. *N-*Myristoylation is a ubiquitous co- and post-translational modification that is necessary for localization and function of several proteins. Moreover, this modification increases the lipophilicity of proteins, facilitating their association with membranes and promoting protein-protein interactions [8]. Myristoylation of several proteins involved in cellular regulation and signal transduction, such as the α-subunit of several G proteins and cAMP-dependent protein kinases, has been shown to be crucial for their function [9,10]. Protein myristoylation has been validated as an essential in *Saccharomyces cerevisiae* [11] and pathogenic fungi, such as *Candida albicans*, *Histoplasma capsulatum*, and *Cryptococcus neoformans* [12]. Consequently, NMT has been explored as an antifungal chemotherapeutic target [13]. Moreover, previous studies have identified NMT as an attractive chemotherapeutic target against protozoan parasites including *Leishmania donovani*, *Leishmania major*, *Trypanosoma brucei*, *and Plasmodium falciparum* [14–16]. More recent detailed studies have validated NMT inhibitors, based on a pyrazole sulfonamide scaffold, for the treatment of sleeping sickness [17], and malaria [18]. In *T*. *cruzi*, few *N*-myristoylated proteins have been experimentally identified; examples include the phosphoinositide-specific phospholipase C and the flagellar calcium-binding protein [19,20]. Nevertheless, bioinformatics analyses have predicted more than 100 proteins to be *N*-myristoylated [21]. In addition, for another post- translational modification, namely palmitoylation, to occur, prior myristoylation is necessary for several important proteins. Palmitoylation, which is the linkage of palmitic acid (C16:0) to cysteine residues, has been shown to be necessary for sorting to the flagellar membrane in kinetoplastids [19,22]. Considering all these facts, it is expected that NMT inhibition would have pleiotropic effects in the physiology of the parasite. Although this enzyme has been extensively characterized in other kinetoplastids [14,16,23,24], very little is known about *T*. *cruzi* NMT (*Tc*NMT). In a recent study by Roberts et al. [25], attempts to generate a double-knockout mutants of *Tc*NMT resulted always in retention of an endogenous copy of the gene. Only in the presence of an ectopic, constitutively expressed NMT copy, was it possible to achieve a double knockout. This strongly suggests that NMT is essential at least for survival of the insect-derived noninfective epimastigote stage of the parasite. Moreover, when epimastigotes were treated with the pyrazole sulfonamide inhibitor **2**, there was a relationship between the levels of NMT expression and the sensitivity of the parasites to this compound. Parasites overexpressing NMT were significantly less sensitive to the compound than WT parasites. Nonetheless, compound **2** was considerably less potent against *T*. *cruzi* epimastigotes in contrast to *T*. *brucei* bloodstream forms, where this compound was curative in the mouse model of human African trypanosomiasis (HAT). There could be several explanations for the differences in potency: differences in the active site of NMT between these two organisms; differences in compound uptake in the different parasites; or differences in the role of NMT in *T*. *brucei* and *T*. *cruzi*.

Recently, inhibitors against *T*. *brucei* NMT (*Tb*NMT) were identified through an initial screening of 62,000 diversity-based compound library. Subsequent chemistry optimization of the screening hit led to potent *Tb*NMT inhibitors that prevented the proliferation of bloodstream form of *T*. *brucei* with a window of selectivity of over 200-fold with respect to proliferation of mammalian cells [17,26,27]. Although these compounds presented high inhibition both, *in vitro* and *in vivo*, against the extracellular bloodstream form of *T*. *brucei*, their effects against intracellular parasites such as *T*. *cruzi* have not been elucidated. In this study, we evaluated the effect of eight of these inhibitors, which exhibited EC_50_ values at the nanomolar range against *T*. *brucei* [17,26], against mammal-dwelling intracellular amastigote and trypomastigote stages of *T*. *cruzi*. Our results validate *Tc*NMT as a potential chemotherapeutic target in Chagas disease. Moreover, we determined the localization and expression of *Tc*NMT throughout the parasite life cycle.

## Materials and Methods

### Chemicals

Unless otherwise indicated, all reagents and solvents used here were of analytical, HPLC, or mass-spectrometry grade from Sigma-Aldrich (St. Louis, MO), or from Thermo Fisher Scientific (Waltham, MA).

### Mammalian cell culture and parasites

LLC-MK2 (green-monkey kidney epithelial cells) and U2OS (human osteocytes) (ATCC, Manassas, VA) cells were culture in high-glucose Dulbecco’s Modified Eagle Medium (DMEM), supplemented with 10% heat inactivated-fetal bovine serum (HI-FBS), at 37°C, under 5% CO_2_ atmosphere. Tissue culture cell-derived trypomastigote forms of *T*. *cruzi* (TCT) (Y strain) (ATCC) were obtained 5 to 9 days after infection of LLC-MK2 monolayers, as previously described [28]. *T*. *cruzi* epimastigotes (Epi) (Y strain) were maintained axenically in liver-infusion tryptose (LIT) medium at 28°C, as previously described [29].

### Purification of intracellular amastigote (ICA) forms

Intracellular amastigote (ICA) forms were purified as described [30,31]. Briefly, 5 x 10^6^ LLC-MK2 cells were seeded in a 150-cm^2^ tissue culture flask (NUNC, Thermo Scientific) and cells were grown for 3–4 days to reach confluency of ~2 x 10^7^ cells per flask. The medium was then replaced with fresh complete medium and host cells were infected with 1 x 10^8^ TCT (multiplicity of infection (MOI) ≈ 5). After 5 days, the infected monolayers were gently detached by scraping and resuspended in 5 mL phosphate-buffered saline (PBS). The suspension containing the infected cells was next transferred to an M-tube (Miltenyi Biotec Inc., San Diego, CA). This tube bears a special stator and rotor that allows for tissue homogenization. In addition, it has a pre-inserted mesh that retains larger particles, removing them from the homogenized sample. The tube was then placed in the gentleMACS Dissociator (Miltenyi Biotec) for the automated disruption of host cells. The homogenized sample containing the parasites was then passed through a column containing anion exchange preswollen microgranular diethylaminoethyl cellulose (DE52, Whatman, Sigma-Aldrich). The sialylated TCT forms of the parasites were retained in the resin, while the ICA forms were recovered at 98–99% yield [30,31].

### Indirect immunofluorescence assay

Parasites were washed with phosphate buffered saline (PBS), pH 7.4, and 1 x 10^4^ cells per well were deposited on a 96-well plate, followed by centrifugation at 3,000 x*g* for 10 min at room temperature. Supernatant was removed and cells were fixed with 4% paraformaldehyde (PFA) for 15 min at room temperature, then permeabilized with 0.1% Triton X-100 in PBS for 10 min. Wells were blocked for 1h at room temperature with 3% bovine serum albumin (BSA) in PBS. To obtain polyclonal anti-TcNMT, rabbit sera was rose against the TcNMT peptide RGDGNLHYYFYNWSYP (Biomatik USA LLC., Wilmington, DE). Anti-TcNMT was labeled with Alexa 594, while rabbit anti-TbBiP (a kind gift from Dr. James Bangs, University at Buffalo, The State University of New York) was labeled with Alexa 488, using the APEX Antibody Labeling Kits (Invitrogen, Thermo Scientific) according to the manufacturer instructions. Parasites were incubated overnight with the primary antibodies, both at 1:250 dilution in 3% BSA-PBS. To remove unbound antibody, cells were washed 3x with PBS with 0.1% Tween (0.1% PBS-T). Parasite DNA was then labeled with 4’,6-diamidino-2-phenylindole (DAPI) (Thermo Scientific) at 0.5 μg/mL. Samples were visualized using a LSM 700 Confocal Microscope (Zeiss) with a 63X oil objective lens. Images were acquired using the ZEN 2009 software (Zeiss).

### Metabolic labeling

Parasites were washed with PBS three times and 1 x 10^7^ cells were incubated at 37°C in the case of ICA and TCT, and at 28°C in the case of Epi, in 1 mL of 2% delipidated BSA-DMEM. After 30 min, 100 μM Click-IT myristic acid, azide (Life Technologies, Thermo Fisher Scientific) was added from 50 mM stock solutions in dimethyl sulfoxide (DMSO). The same volume of DMSO was used as a negative control. Parasites were further incubated for 6 h. For confocal microscopy analysis, they were processed as described above. After permeabilization (0.1% Triton X-100 in PBS for 10 min, at room temperature), cells were washed three times with 3% BSA in PBS. “Click” reaction between the myristic acid azide and Alexa Fluor 488 alkyne (Invitrogen) was performed according to the manufacturer instructions using the Click-iT Cell Reaction Buffer Kit (Invitrogen). Parasite DNA was labeled with DAPI. Samples were visualized and images acquired as described above.

### In vitro parasite proliferation assay

Compounds **1–8** were synthesized at the University of Dundee Drug Discovery Unit [17,25–27,32]. These DDD compounds and the reference drug benznidazole (BZ) (Sigma-Aldrich, St. Louis, MO) were prepared 6-fold-concentrated in 6% DMSO, to give a final concentration of 1x compound in 1% DMSO. Thirty microliters of each DDD compound were added to a 96-well microplate (Nunc, Thermo Scientific) to give the final concentrations of 50, 10, 1, 0.1, 0.01, and 0.001 μM, whereas BZ was added at the final concentrations of 800, 400, and 40 μM. DMSO control wells contained 30 μL 6% DMSO. Human osteocytes (U2OS cells) were diluted in DMEM, 10% heat inactivated-fetal bovine serum (HI-FBS) to give a cell density of 2 x 10^5^ cells/mL. *T*. *cruzi* trypomastigotes were resuspended in DMEM, 10% HI-FBS to a final density of 1.2 x 10^7^. Cells and parasites were mixed a ratio of 4 volumes of cells plus 1 volume of parasites. 150 μL of the mixture were dispensed per well. Plates were incubated for 48 h. After the incubation period, media was removed from the wells and 100 μL 4% paraformaldehyde (PFA) were added. After 15 min of incubation at room temperature, PFA was removed and cells and parasites were stained with Draq5 (BioStatus, Leicestershire, UK) at a concentration of 5 μM diluted in PBS. The plates were then read in a bioimager system as described below. Three independent experiments were performed in triplicate wells.

### High-content imaging (HCI) assay

Image acquisition and analyses of the plates were carried out using the BD Pathway 855 high-resolution fluorescence bioimager system (BD Biosciences, San Jose, CA), as previously described [33]. Filter sets appropriate for the excitation and emission spectra of Draq5 were utilized. Images from four fields (2 x 2 montage) were acquired per well with a 20x objective. To perform the host cell segmentation and counting of parasites, the BD AttoVision v1.6.2 Sub Object analysis was used. Draq5 creates a background, staining the host cell and parasite nucleus, but defining the cytoplasm as well, determining this way the amount of parasites within each mammalian cell. The host cell nucleus was excluded by size difference. Raw data was imported into the BD Image Data Explorer (BD Biosciences) to determine the percentage of infected cells containing at least 1, at least 3 or at least 5 intracellular parasites.

### Cytotoxicity assay

DDD compounds **1–8** and the reference drug BZ were prepared 6-fold-concentrated in 6% DMSO, to give a final concentration of 1x compound in 1% DMSO. Thirty microliters of each compound were added to give the final concentrations of 50, 10, 1, 0.1 and 0.01 μM. DMSO control wells contained 30 μL 6% DMSO, whereas wells for the negative control contained 1% hydrogen peroxide in DMEM. U2OS cells were resuspended in DMEM, 10% HI-FBS, to give a cell density of 2 x 10^5^ cells/mL, while ICA forms were resuspended in DMEM, 10% HI-FBS, to a final density of 1 x 10^7^ per well. Epi forms were resuspended in LIT medium to a density of 1 x 10^6^ parasites per well. Hundred and fifty microliters of the suspension containing either host cells or parasites were added per well. Following 47- and 23-h incubation at 37°C for mammalian cells and ICA, respectively, a mixture of propidium iodide (Invitrogen) and Hoechst dye (Invitrogen) diluted in culture media (1 μg/mL each) was added to the plates, which were then further incubated for 1 h at 37°C. Finally, plates were read in the BD Pathway 855 bioimager system. In the case of Epi, after 68-h incubation at 28°C, 18 μL Alamar Blue (AbD Serotec, Raleigh, NC) were added per well. Following an additional 4-h incubation, the plate was read in a microplate fluorometer (Thermo Scientific) at excitation 560 nm, emission 590 nm. Three independent experiments were performed in triplicate wells.

### Determination of EC_50_

The half maximal effective concentrations (EC_50_) were determined using GraphPad software (GraphPad Software, Inc., La Jolla, CA) using a sigmoidal dose-response variable slope model with response values based on the total number of parasites or cells (normalized to span de range from 0 to 100%) plotted against the logarithm of compound concentration. EC_50_ were determined from three independent experiments.

### In-gel western blotting

Parasites were incubated with or without 10 μM of the inhibitor **1**, **5**, or **8** in 1 mL 2% delipidated BSA-DMEM at 37°C for ICA and TCT and at 28°C for Epi. After 6 h, 100 μM myristic acid, azide (from a 50-mM stock in DMSO) were added to the samples, and the same volume of DMSO was added to the negative control. Cells were incubated for another 6 h, for a 12-h treatment with the inhibitors. Next, the parasites were washed three times with PBS and lysed in 1% SDS, Tris-HCl, pH 8.0. “Click” reaction was performed between the myristic acid azide and Biotin Alkyne (Invitrogen) according to the manufacturer instructions using the Click-iT Protein Reaction Buffer Kit (Invitrogen). Duplicate aliquots of lysates were ran in 10% SDS-PAGE followed by fixing (5% acetic acid, 50% isopropanol) for 15 min. Gels were then analyzed by in-gel western blotting using IRDye streptavidin (LI-COR Biosciences, Lincoln, NE) 1:7500 in 5% BSA, 0.05% SDS, 0.2% Tween in PBS, for 1 h at room temperature. They were subsequently treated or not with 0.2 M NaOH in methanol for 1 h at room temperature. Gels were scanned in an ODYSSEY quantitative imagining system (LI-COR Biosciences). They were next stained with Coomassie blue and scanned again to determine total protein levels.

### [^35^S]-Methionine labeling

Parasites were incubated with or without 10 μM of the inhibitors as described above. After 10 h, cells were starved in methionine deficient media for 1 h. Next, 20 μCi.mL^-1^ [^35^S]-methionine (Perkin Elmer) were added to the samples for 1 h incubation, for a 12-h treatment with the inhibitors. Next, the parasites were washed three times with PBS and lysed in 1% SDS, Tris-HCl, pH 8.0. Samples were ran in 4–12% gels (Expedeon, San Diego, CA) and transferred to polyvinylidene fluoride (PVDF) membranes (Sigma-Aldrich). Membranes were dried and exposed to Hyperfilm ECL (Amersham, GE Healthcare Lifesciences, Piscataway, NJ). To determine total protein levels, membranes were stained with Ponceau S after exposure.

### Western blotting analysis

Parasites (1 x 10^7^ cells) were incubated with or without 10 μM of the inhibitor (compound **1**, **5**, or **8**) in 1 mL 10% HI-FBS, DMEM at 37°C for ICA and TCT, and at 28°C for Epi for 12 h. Next, parasites were washed three times with PBS and lysed in 1% SDS, Tris-HCl, pH 8.0. Samples were run in 10% SDS-PAGE followed by western blot probed with anti-*Tc*NMT at dilution 1:1000 in 5% milk-PBS overnight. This was followed by incubation with anti-rabbit IgG-HRP conjugated. The membrane was next stripped with boiling stripping buffer (3% SDS, 0.3% β-mercaptoethanol) for 10 min, followed by 4 washes with 0.1% PBS-T. It was then blot again with anti-BiP at dilution 1:500 in 5% milk-PBS overnight, at 4°C, followed by anti-rabbit IgG HRP-conjugated.

## Results

### NMT expression and localization in *T*. *cruzi*

**Fig 1:**
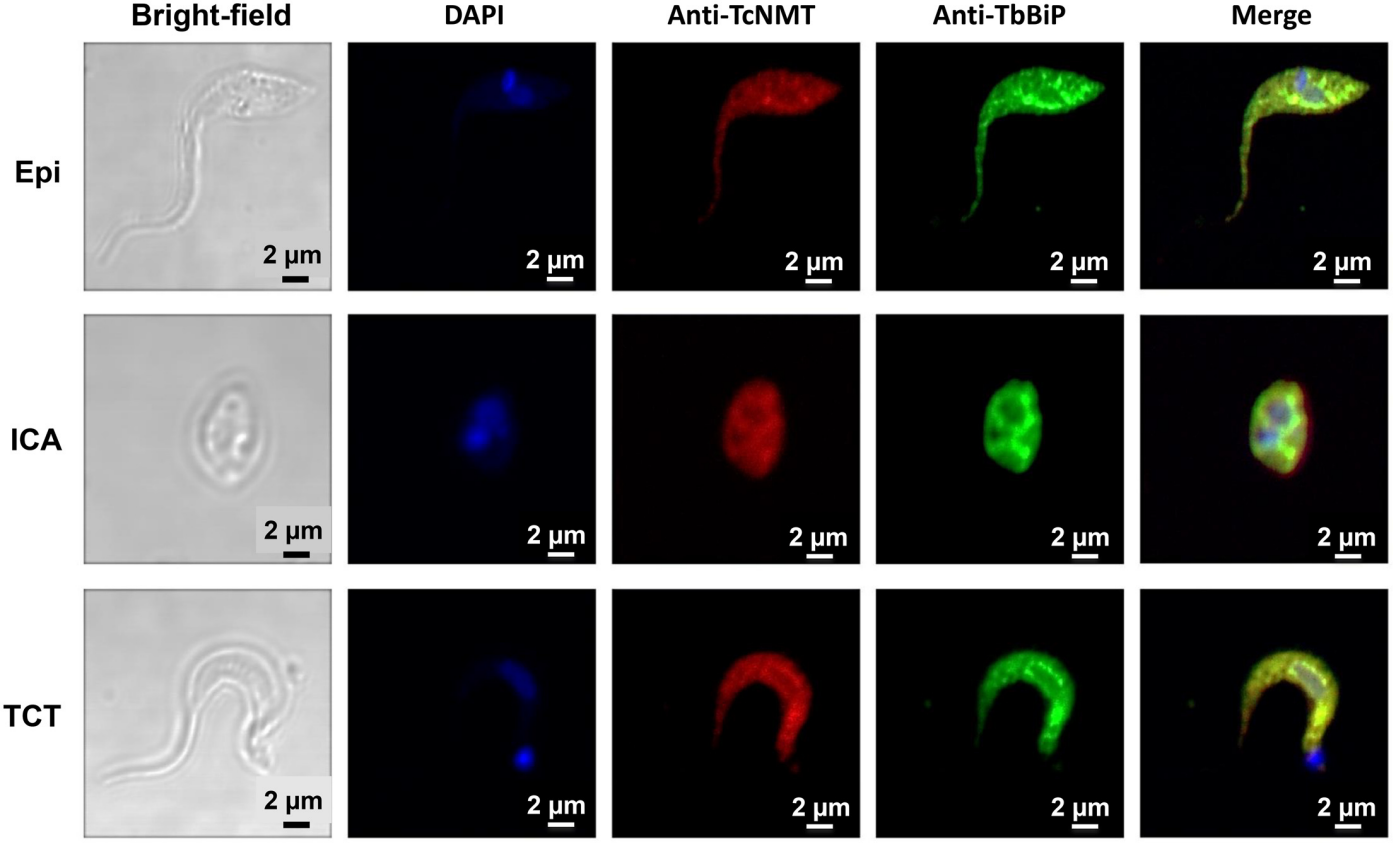
TcNMT is constitutively expressed in all *T*. *cruzi* stages and it is associated to the endoplasmic reticulum. Immunofluorescence microscopy of Epi, ICA, and TCT. Cells are shown as viewed under phase contrast, visualized for fluorescence with anti-TcNMT (red) and anti-BiP (green), co-stained with DAPI to reveal positions of the nucleus and kinetoplast (blue). Scale bar, 2 μm.

**Fig 2:**
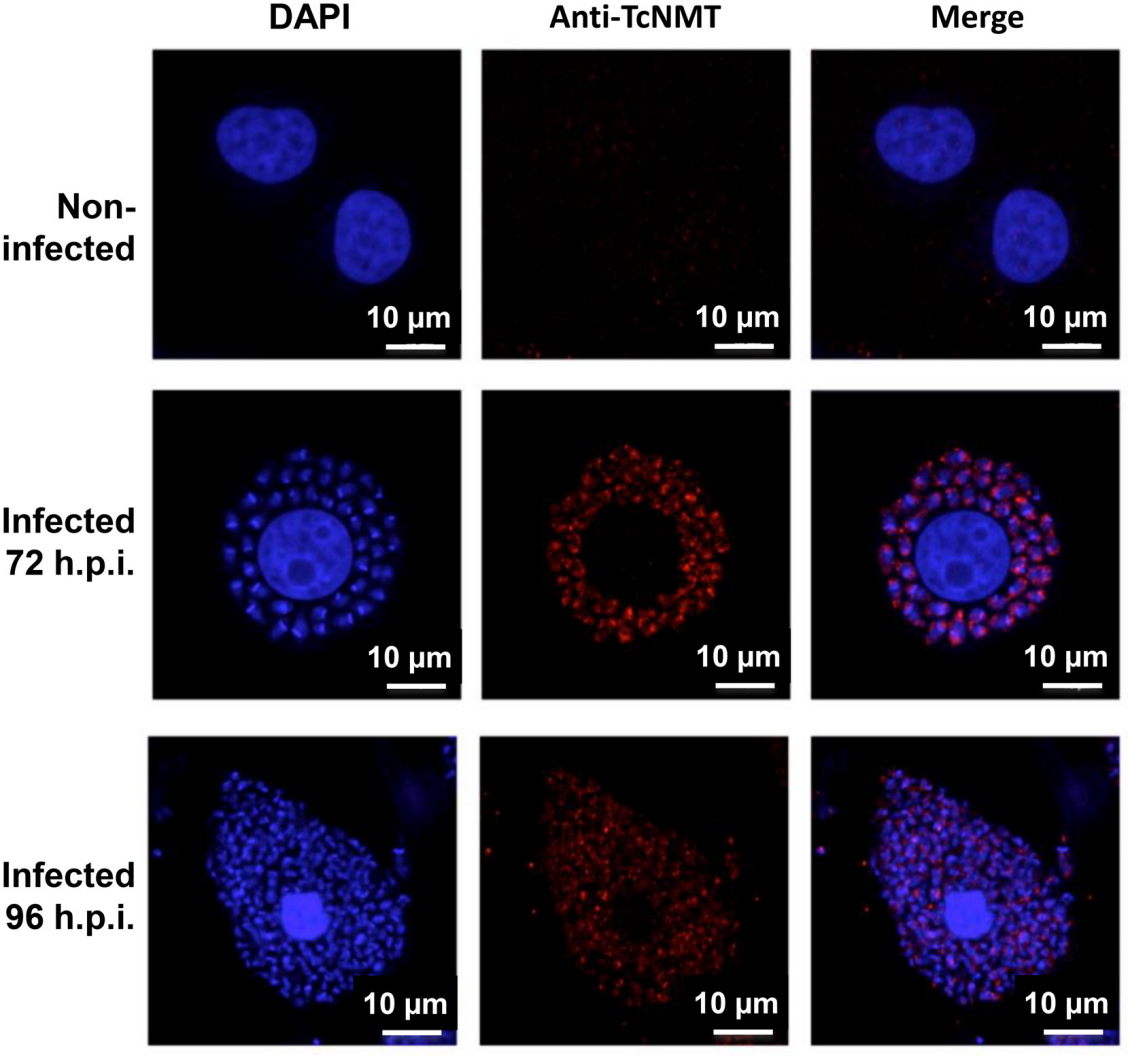
Anti-TcNMT shows no cross-reactivity with human cells. Immunofluorescence microscopy of non-infected and infected cells 72- and 96-h post-infection stained with anti-TcNMT (red), co-stained with DAPI (blue) to visualize host cell and parasite DNA. Scale bar, 10 μm.

To investigate the expression and cellular localization of *Tc*NMT during the *T*. *cruzi* life cycle, epimastigote (Epi), intracellular amastigote (ICA), and tissue cell culture-derived trypomastigote (TCT) forms were analyzed by immunofluorescence, using an antiserum (anti-*Tc*NMT) obtained in rabbit against the *Tc*NMT peptide RGDGNLHYYFYNWSYP. Anti-*Tc*NMT showed that NMT has a punctate pattern in Epi and TCT and more diffused labeling in ICA. In all three stages, however, there is little or no labeling localized to the nucleus and kinetoplast. The results indicate that the anti-*Tc*NMT labeling is mostly associated with the endomembrane system and other intracellular sites in the parasite. To further determine the subcellular localization of *Tc*NMT we use anti-*Tb*BiP as an endoplasmic reticulum (ER) marker [34]. Colocalization with BiP suggests that NMT is at least partially associated to the ER of *T*. *cruzi* (Fig 1). Taken together, these results demonstrate that *Tc*NMT is constitutively expressed in all the stages of the *T*. *cruzi* life cycle, as previously shown by Roberts et al. using immunoblots of crude lysates [25], and that it is associated with the ER. To determine the specificity of anti-*Tc*NMT, human osteocytes were infected with *T*. *cruzi* and analyzed by immunofluorescence. Anti-*Tc*NMT readily labeled intracellular parasites at 72-h (ICA) and 96-h (mostly TCT) post-infection, whereas non-infected cells were not labeled, indicating that there is no cross-reactivity between this antiserum and human cells (Fig 2).

*Tc*NMT shares 59% and 57% overall amino acid identity with NMTs from *T*. *brucei* and *L*. *major*, respectively. Antiserum against *Lm*NMT and antiserum against *Tb*NMT also recognized *Tc*NMT in Epi (S1 Fig). The expression and distribution of *Tc*NMT is consistent with that observed in *L*. *major* and *T*. *brucei* [23,24], where NMT is also expressed in both the insect-vector and the mammalian forms of the parasites. Sequence analysis showed that similarly to *Tb*NMT and *Lm*NMT, *Tc*NMT is divergent in its N-terminus from all NMTs so far characterized. Interestingly, even though our data suggest ER association, *Tc*NMT does not contain the lysine-rich regions that have been associated with ribosomal targeting in human and mouse NMTs [35].

**Fig 3:**
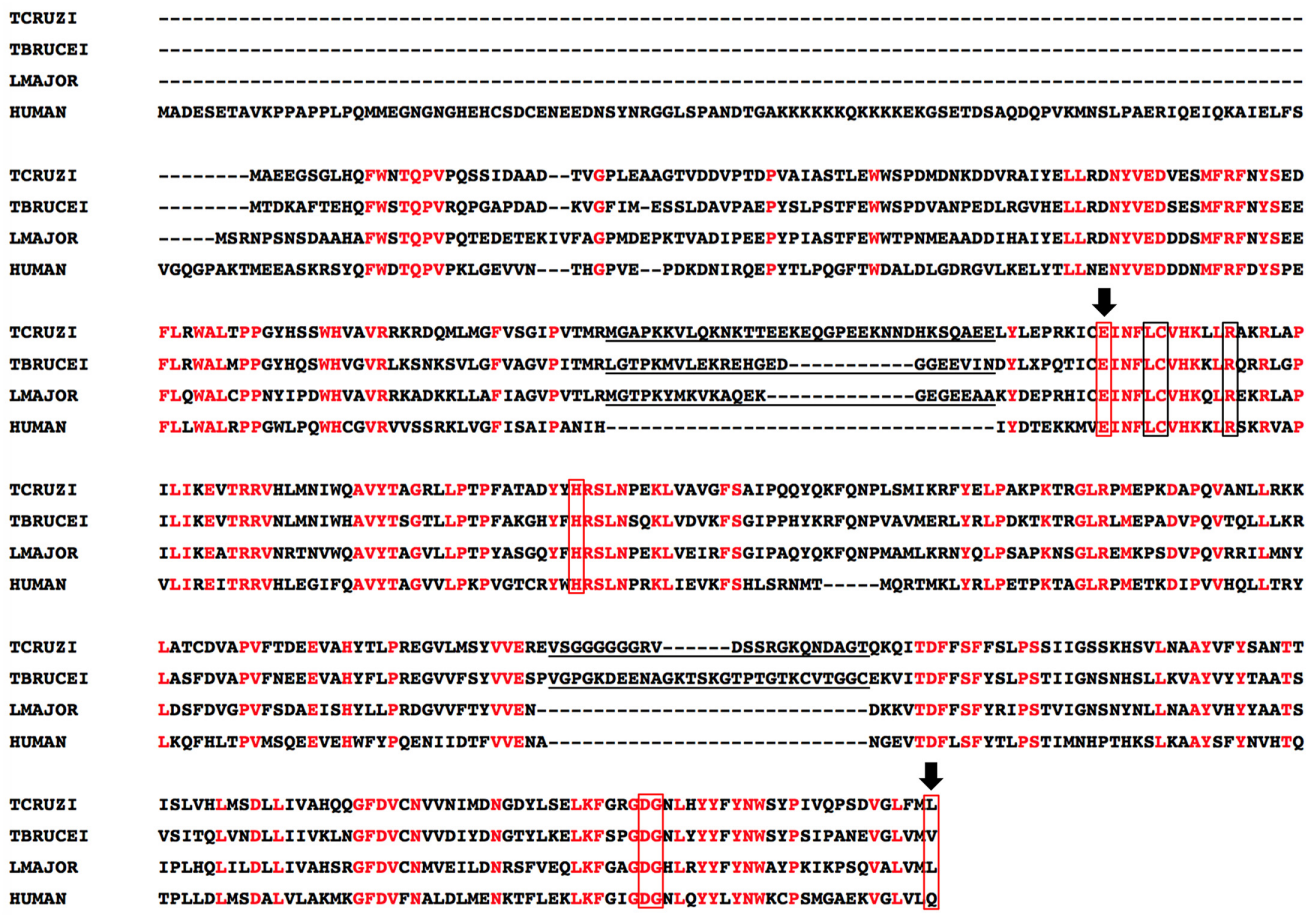
Alignment of *T*. *cruzi* NMT with NMTs from *T*. *brucei*, *L*. *major* and human. The deduced open reading frame of *Tc*NMT (AI069625) was aligned with *Tb*NMT (TRYP10.0.001826–6), *Lm*NMT (AF3059561), and human NMT (*Hs*NMT) (*HUMAN*, P30419) using the ClustalW2 multiple sequence alignment program (http://www.ebi.ac.uk/Tools/msa/clustalw2/). Strictly conserved residues are shown in red. The insertions in protozoan NMTs (*Tc*NMT, *Tb*NMT, and *Lm*NMT) are underlined. Red boxes indicate key residues involved in myristoyl-CoA binding; black boxes indicate residues involved in peptide binding identified in yeast species. Arrows identify the pocket floor residues in *C*. *albicans*.

NMT residues that have been predicted to be essential for the activity of this enzyme, including the two negatively charged residues, Leu 452 and Glu 173, that have been predicted to form the floor of the active site pocket in *C*. *albicans* NMT [6,36], are conserved in *Tc*NMT. An insertion of 22- or 20-mer is found close to the first “pocket floor” in *Tb*NMT and *Lm*NMT, respectively, relative to human and fungal NMTs [23]. In the same region, between the residues Arg 129 and Leu 164, a 34-mer insertion is observed in *Tc*NMT, but completely absent in human NMT (*Hs*NMT). An additional insertion in *Tc*NMT (between Glu 324 and Gln 347) and *Tb*NMT is found further downstream, but completely absent in *Lm*NMT and *Hs*NMT. These differences between protozoan, fungal, and human NMTs suggest that the enzyme activity might be affected by additional secondary or tertiary structures formed in these parasites NMTs relative to human and fungal enzymes (Fig 3).

### Myristic acid azide-labeled proteins are associated with the endomembrane system

**Fig 4:**
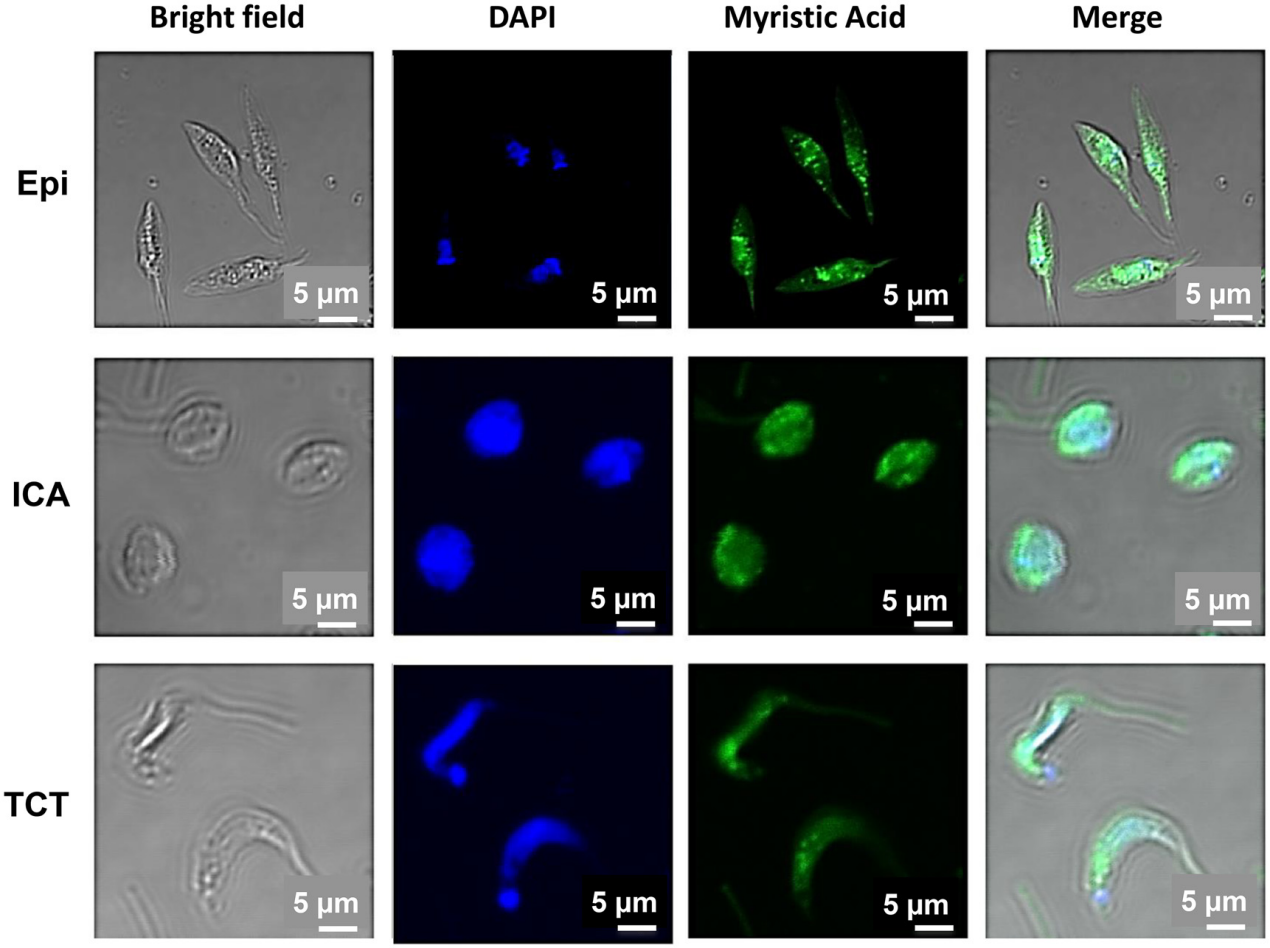
Myristoylated proteins are associated to the endomembrane system. Fluorescence microscopy of Epi, ICA, and TCT metabolically labeled with myristic acid, azide were reacted with Alexa 488 alkyne (green) after permeabilization, co-stained with DAPI (blue) to label the nucleus and kinetoplast. Scale bar, 5 μm.

It has been shown that metabolic labeling with azido-fatty acid analogs followed by “click” reaction with an alkyne-containing fluorophore, results in fluorescently labeled fatty-acylated proteins [37]. To visualize the distribution of *N-*myristoylated proteins we first labeled Epi, ICA, and TCT with myristic acid, azide. This azide tag is small enough to permit the incorporation of the tagged myristic acid molecule onto proteins by NMT and has recently been shown to efficiently label N-myristoylated proteins in *T*. *cruzi* Epi forms [25]. Previous studies have shown that treatment with Triton X-100 solubilizes and extracts fatty acid chemical reporters that are not covalently attached to proteins [37]. After fixing, we then permeabilized parasite cells with Triton X-100 and performed chemoselective ligation or “click” chemistry between the azide and Alexa Fluor 488 alkyne. By following this protocol, we could ensure that the majority of the Alexa Fluor 488 signal corresponds mostly to putative *N-*myristoylated proteins. The confocal microscopy showed mainly a punctate pattern in all three *T*. *cruzi* life-stage forms, indicating endomembrane association (Fig 4). Overall, these results are consistent with the notion that *N-*myristoylation targets proteins to membranes [38].

### DDD compounds inhibit *T*. *cruzi* intracellular proliferation

**Fig 5:**
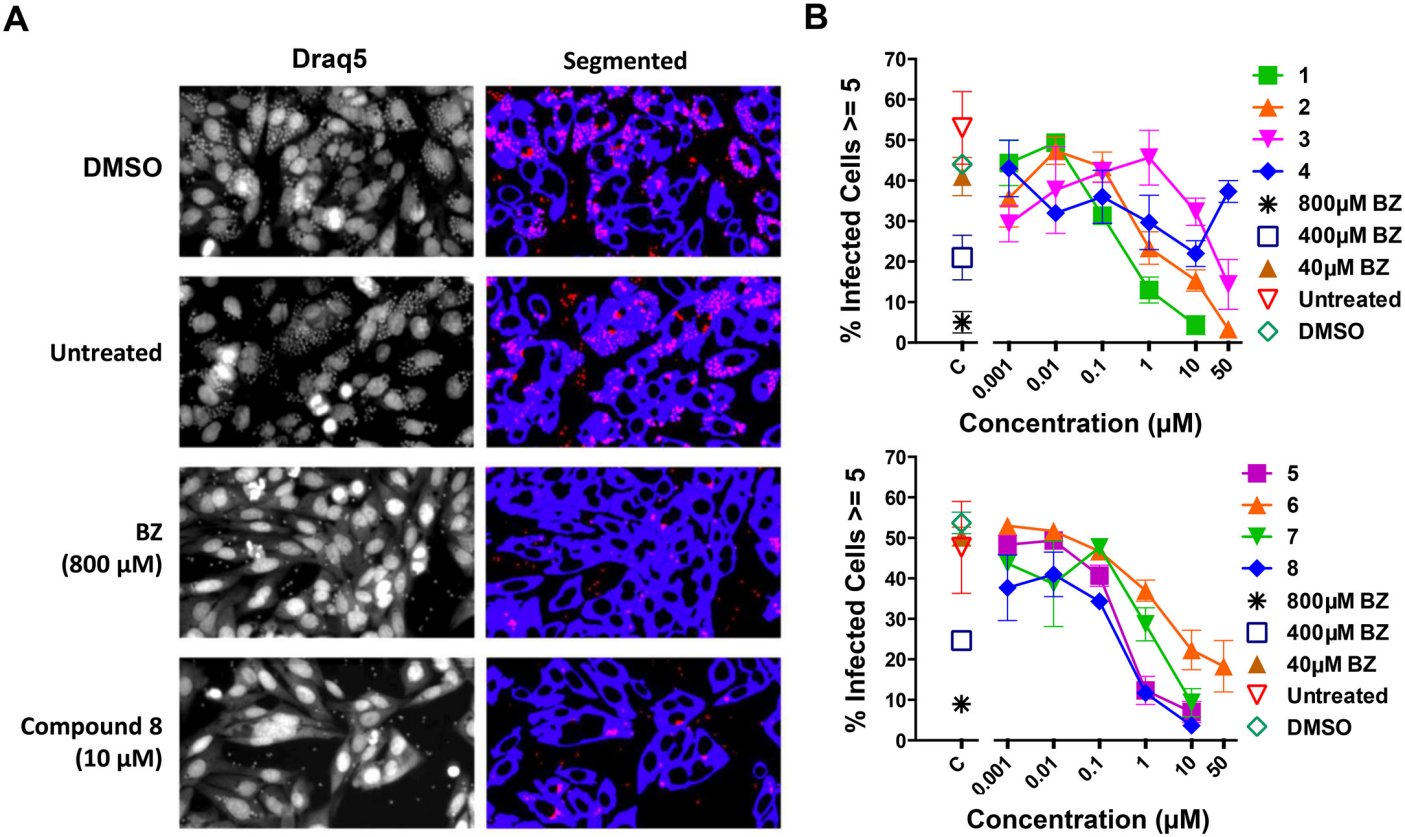
DDD compounds inhibit intracellular proliferation of *T*. *cruzi*. (**A**) Representative images of cells treated with the vehicle control (DMSO), untreated, treated with 800 μM benznidazole (BZ) or 10 μM compound **8**, stained with Draq5 (left panel), and analyzed by HCI. Artificial images created after segmentation on the fluorescence bioimager (right panel). Host cells are shown in blue, extracellular parasites in red and intracellular parasites in pink. (**B**) The multiparametric data obtained on a cell-by-cell basis by HCI was analyzed to determine several parameters associated to infection of host cells by *T*. *cruzi* including the percentage of cells infected with at least five parasites (percentage of cells in which the parasite proliferated), treated or not with DDD compounds **1–8**. C, controls: 40, 400, and 800 μM BZ, DMSO, and Untreated.

**Fig 6:**
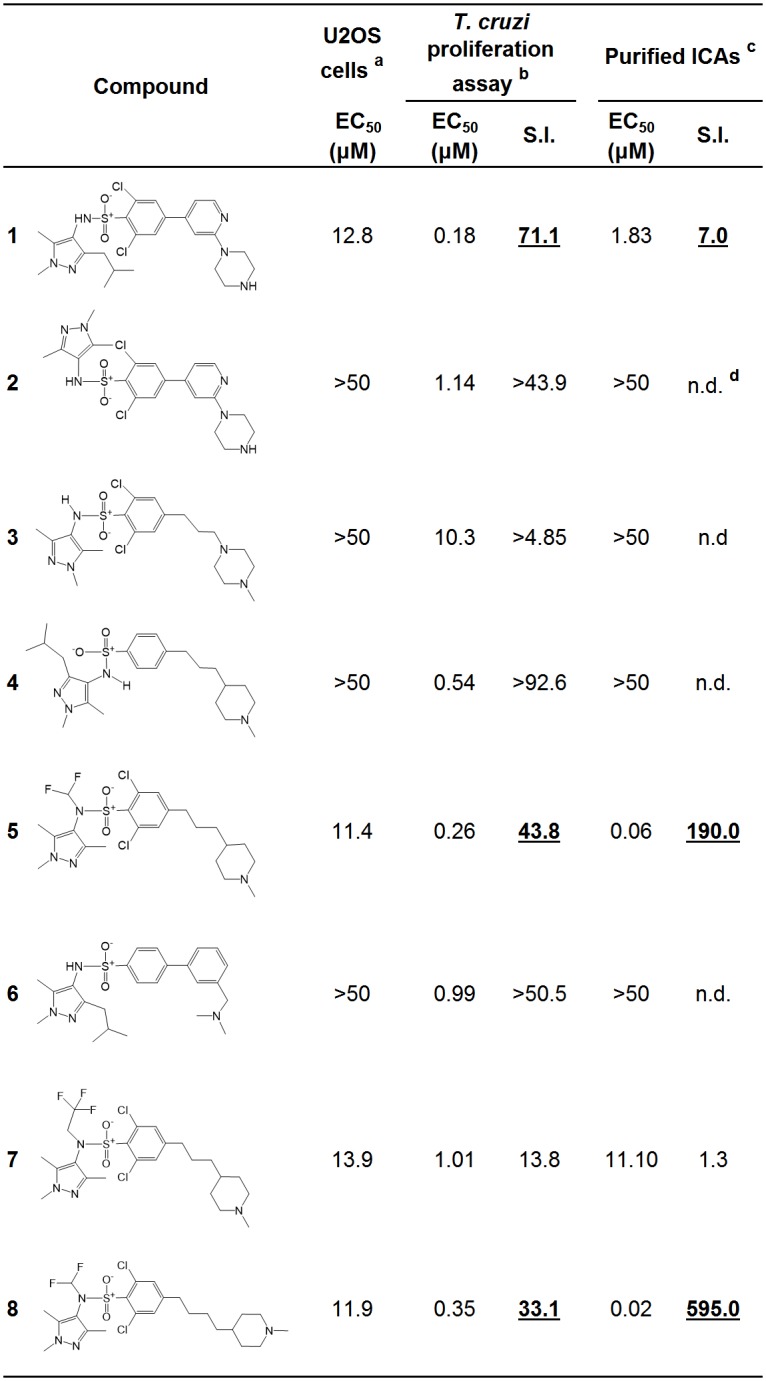
EC_50_ values and selectivity index (S.I.) of DDD compounds in noninfected and *T*. *cruzi*-infected U2OS cells, and purified intracellular amastigotes. (**a** and **b**) Non-infected and infected U2OS cells, respectively, were incubated for 48 h at 37°C with DDD compounds 1–8. (**c**) Purified ICAs were incubated for 24 h at 37°C with DDD compounds 1–8. (**d**) Not determined.

Previous studies demonstrated the efficacy of the studied pyrazole sulfonamides as NMT inhibitors against *T*. *brucei* infective bloodstream forms [17,26] and *T*. *cruzi* noninfective Epi forms [25]. However, there have been no reports on the activity of these compounds against the human-disease relevant (ICA and TCT) stages of *T*. *cruzi*. Here, we tested selected NMT inhibitors to determine their effects against the intracellular proliferation of *T*. *cruzi* in human osteocytes (U2OS cells). Draq5 stains nucleic acids; however, it creates a background defining the cytoplasm of the host cell. Therefore, it stains the nucleus of the host cell and the parasite with a higher intensity, while staining the cytoplasm of the mammalian cell with a fainter intensity, rendering the software able to distinguish one from the other. An example of the segmentation performed on the fluorescence bioimager is shown in Fig 5A. Upon entering the cell, trypomastigotes differentiate into replicative amastigote forms, which then begin to divide. Approximately 96 h after infection the parasite transforms again into trypomastigotes, finally rupturing the cell and releasing the parasites into the extracellular milieu [39]. Since we are interested in the antiproliferative effects of the compounds, we incubated for 48 h prior to fixation. At this time point, significant replication of the parasite is observed with little host-cell disruption. To determine the percentage of infected cells, we evaluated cells containing at least one parasite (S2A and S2B Fig). We did not see significant differences in the number of infected cells between the untreated wells and the wells treated with the compounds below 10 μM concentration. To determine the proliferation inside the mammalian cells, the number of cells containing at least 3 (S2C and S2D Fig) or 5 (Fig 5B) parasites was plotted. Compounds **1**, **5** and **8** were able to inhibit intracellular amastigote proliferation at submicromolar concentrations, with EC_50_ values of 0.18 μM, 0.26 μM, and 0.35 μM, respectively, with selectivity index (S.I.) of 71, 44, and 33, respectively (Fig 6; bold, underlined numbers). Although compounds 4 and 6 also exhibited submicromolar EC_50_ values (0.54 and 0.99 μM, respectively) and good S.I. (>93 and >51, respectively) in the *T*. *cruzi* proliferation assay, they were not effective against purified ICAs (EC_50_>50 μM) (Fig 6). Therefore, they were not further analyzed here as potential anti-*T*. *cruzi* agents.

### DDD compounds are trypanocidal and nontoxic to mammalian cells

**Fig 7:**
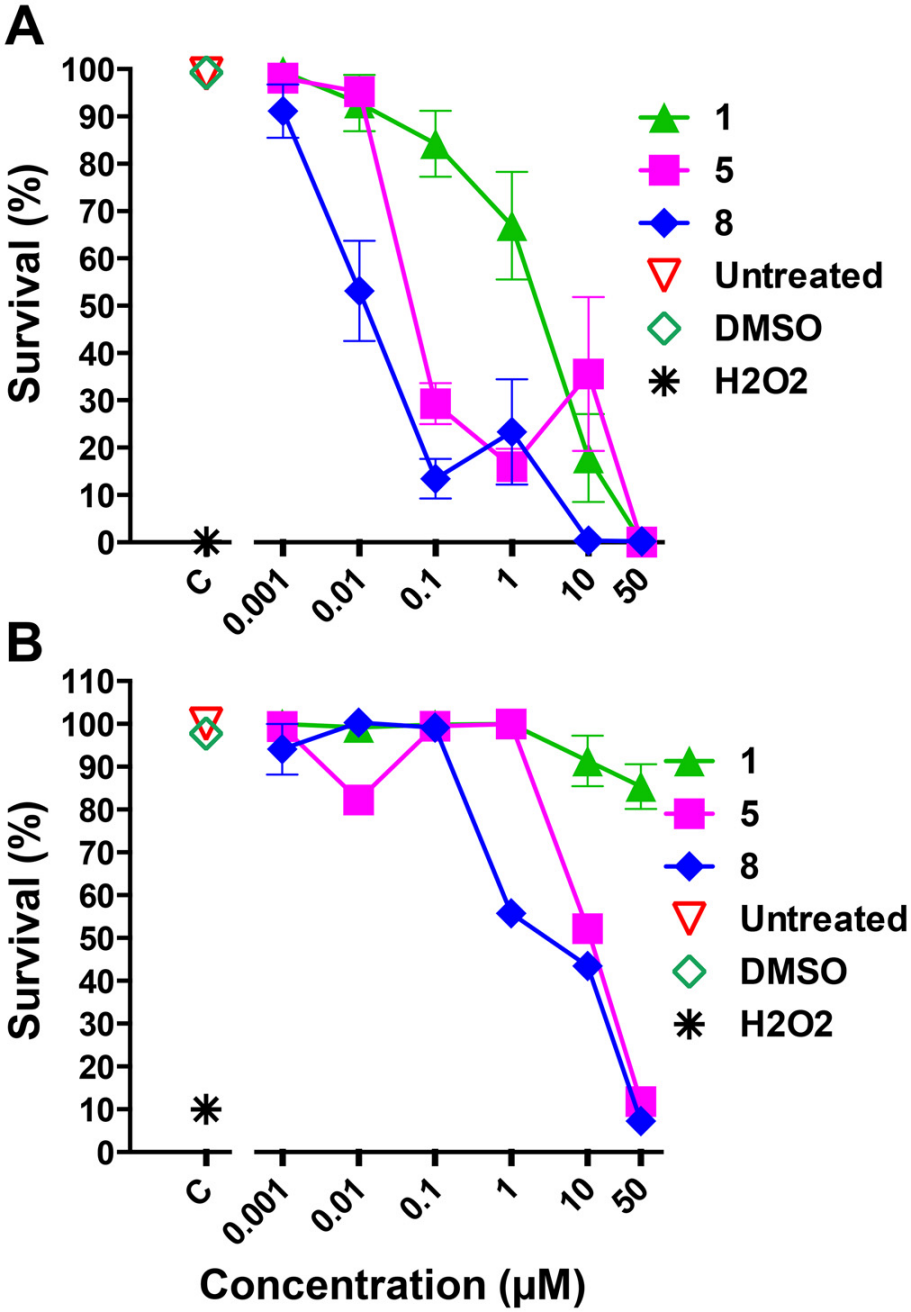
DDD compounds are trypanocidal against purified intracellular amastigotes. Total number of parasites in each well was counted to evaluate the cytotoxicity of the compounds **1**, **5**, and **8** against purified ICA (**A**) and Epi (**B**). C, controls: Untreated, DMSO, and H_2_O_2_.

These compounds were originally synthesized to target an extracellular parasite, namely *T*. *brucei*. Therefore, we wanted to determine if the cytotoxic effects against the intracellular parasite *T*. *cruzi* could be increased once the barrier of host cell membrane was not present. If compound access to the parasites proved problematic, understanding what properties in the compound are important for access to the intracellular parasites would assist compound design and selection. To determine the effects directly on ICA forms, we purified them from the host cells and incubated them with the inhibitors for 24 h. As expected, compounds **1**, **5**, and **8**, which were the most effective at inhibiting proliferation, were also the most cytotoxic against purified ICA forms, exhibiting an EC_50_ of 1.8 μM, 0.06 μM, and 0.02 μM, respectively (Figs 6 and 7A). We were able to see a more potent effect of the inhibitors **5** and **8** when tested directly against ICA. This suggests that by improving the translocation of these two compounds into the mammalian host cell, the potency of the inhibitors against the intracellular parasites would be enhanced. Compound **2** had shown high efficacy against *T*. *brucei*, both *in vitro* and *in vivo* [17]. Interestingly, in *T*. *cruzi* this compound exhibited an EC_50_ of 1.1 μM (Fig 5B) in the proliferation assay and showed no significant trypanocidal activity against purified ICA forms (S3 Fig). Intriguingly, the Epi forms were less susceptible to these compounds. No significant difference was observed on the viability of the Epi forms of the parasite when increasing the length of the treatment from 48 to 72 h. After 72 h, compound **1** did not have a significant effect on parasite viability, whilst compounds **5** and **8** showed an EC_50_ of 9.7 μM and 2.1 μM, respectively (Fig 7B).

To determine the cytotoxic effects of these compounds on mammalian cells, we performed a viability assay on U2OS cells. Using high-content imaging, we were able to differentiate dead cells by the uptake of propidium iodide. The EC_50_ on U2OS of all three compounds were above 10 μM (Fig 6). After 48 h of treatment, compounds **1**, **5**, and **8** showed toxicity only at 50 μM, while there was no significant effect at lower concentrations (S4 Fig). Since almost complete inhibition of intracellular proliferation of the parasite was observed at concentrations as low as 10 μM, there is a window of selectivity between parasite and mammalian cells (Fig 6).

### Inhibitors act “on target”

**Fig 8:**
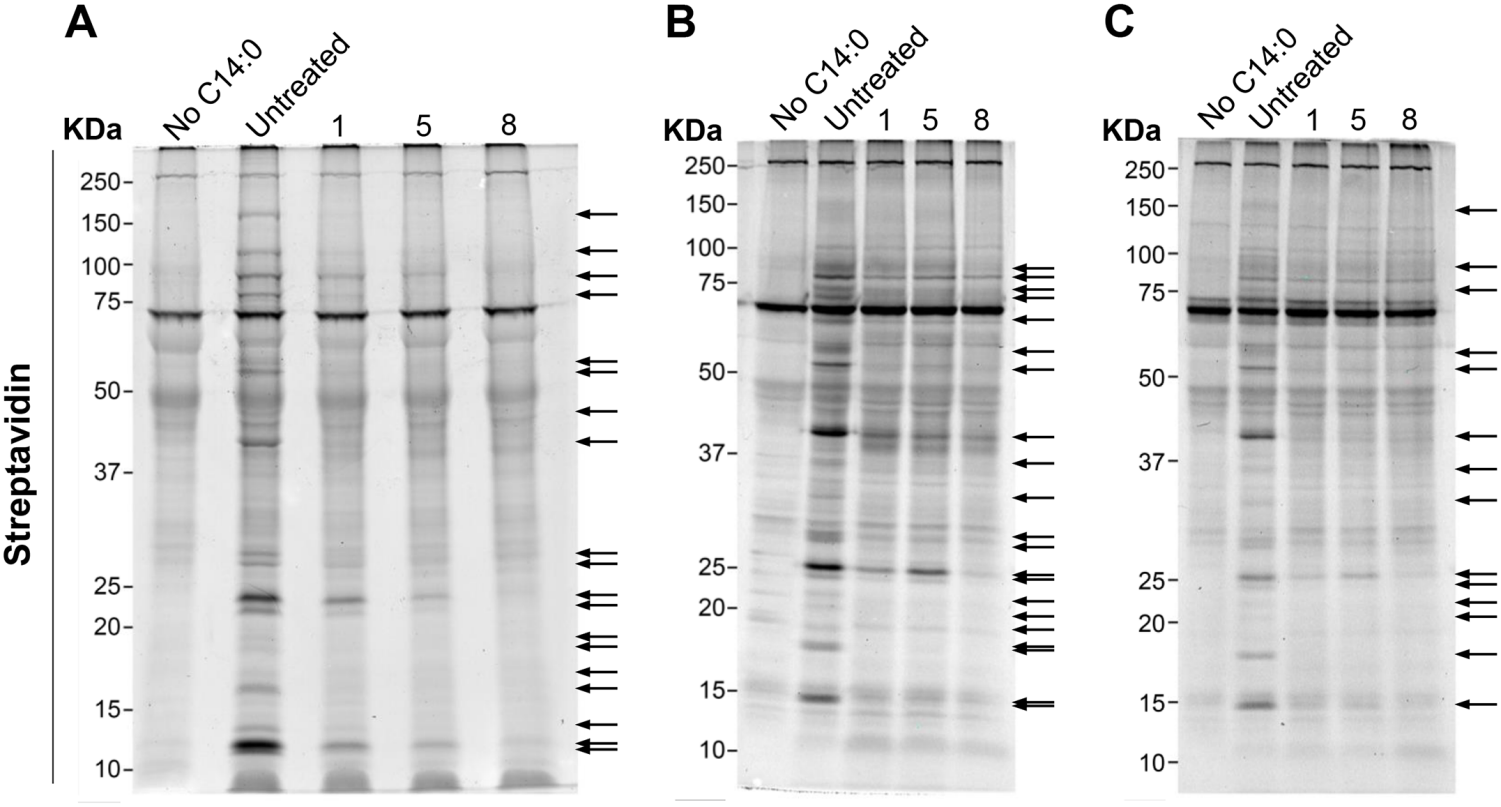
DDD compounds specifically inhibit TcNMT. In-gel western blots of lysates of (**A**) Epi, (**B**) ICA, and (**C**) TCT treated or not with 10 μM of the DDD inhibitor **1**, **5**, or **8**, labeled with myristic acid, azide followed by “click” reaction with biotin alkyne. IRDye 800CW streptavidin was used to detect myristoylated proteins. Arrows indicate protein bands whose labeling was more affected by any of the DDD compounds tested.

To determine whether these inhibitors are acting on *Tc*NMT, we labeled Epi, ICA and TCT forms with myristic acid azide, which had been previously incubated with or without 10 μM of each inhibitor. Detection of *N-*myristoylated proteins is typically performed by metabolic labeling with radioactive [^3^H]-myristate [17]. Alternatively, here we used a biorthogonal labeling method. In the first step, an azido-myristic acid analog was actively incorporated into the parasites. In the second step, the “click” reaction, the azide-modified proteins reacted with a chemoselective alkyne-biotin. After SDS-PAGE and in-gel western blot analysis with IRDye 800CW Streptavidin, several putative *N-*myristoylated proteins were visualized in Epi, ICA, and TCT (Fig 8, Untreated). To confirm that these proteins were indeed *N*-myristoylated a representative duplicate gel of Epi lysate was treated with 0.2 M NaOH in methanol to remove any base-labile hydroxy- or thioester-linked myristic acid azide. Treatment of putative Epi *N-*myristoylated proteins with base prior to scanning showed no difference in the number and intensity of bands indicating that they are *N-*myristoylated proteins (S5 Fig). Two bands, at approximately 70 and 250 kDa, were present in all samples from Epi, ICA, and TCT (Fig 8). They probably represent proteins that are endogenously biotinylated; such as the biotin-dependent carboxylases, 3-methylcrotonyl-CoA and acetyl-CoA carboxylase which have been extensively characterized in *T*. *brucei* [40]. Incubation of the parasites with 10 μM of compound **1**, **5**, or **8** for 12 h resulted in either a decreased intensity or complete loss of most of the bands in all the stages of the parasite (Fig 8). To determine whether these compounds specifically inhibited *N-*myristoylation, parasites from the three different stages of *T*. *cruzi* were labeled with [^35^S] methionine [17]. Treatment with compound **1**, **5**, or **8** had no effect on the incorporation of [^35^S]-methionine into proteins (S6 Fig), indicating that the inhibitors do not affect protein translation. Taken together, these results suggest that the DDD compounds specifically inhibit *N-*myristoylation in all *T*. *cruzi* stages and that this is directly linked to inhibition of proliferation.

### Epimastigotes treated with DDD compounds overexpress NMT

**Fig 9:**
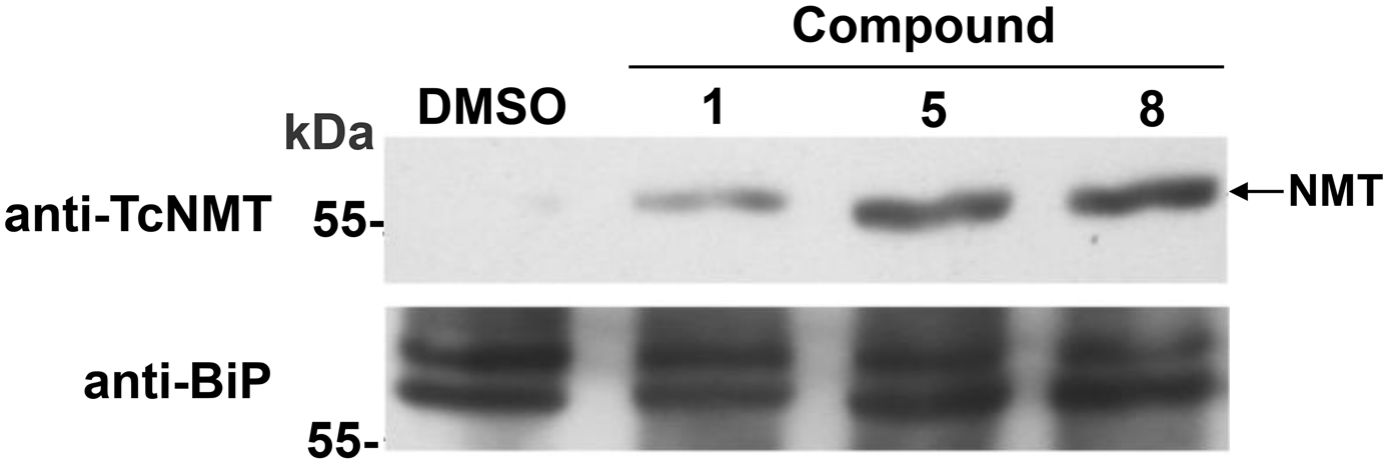
TcNMT is overexpressed in epimastigotes treated with DDD compounds. Epi forms were treated for 12 h with or without 10 μM of compound **1**, **5**, or **8**. Levels of NMT expression were confirmed by western blotting using anti-TcNMT. BiP (binding protein) was used as a loading control.

To investigate the expression and cellular localization of *Tc*NMT in parasites treated with the compounds, Epi, ICA, and TCT were analyzed by western blotting after 12 h of treatment with 10 μM of compound **1**, **5**, or **8**. *Tc*NMT in lysates from wild-type parasites could not be detected by western blotting, probably due to low expression in all stages of *T*. *cruzi*. However, Epi treated for 12 h with 10 μM of compounds showed an overexpression of *Tc*NMT that was detectable (Fig 9). We were unable to see the same phenomenon in lysates from ICA and TCT forms pre-incubated with the DDD compounds. Moreover, these observations may explain the increased resistance of Epi to the inhibitors (Fig 7B). Even though we still see a reduction in protein *N*-myristoylation in Epi (Fig 8), the overexpression of *Tc*NMT might be enough to compensate for the inhibitory effects of the compounds, rendering these parasites less susceptible to the treatment. Encouragingly, these data further confirm the “on-target” effect of these compounds.

## Discussion

Despite the impressive advances in understanding the protozoan parasite *T*. *cruzi*, Chagas disease continues to cause significant morbidity and mortality. The two drugs available to treat this disease (i.e., benznidazole and nifurtimox) are decades old, limited in efficacy, and present severe side effects. Moreover, there is no vaccine available. Chagas disease is therefore classified as a neglected tropical disease. Accordingly, there is an urgent need for new chemotherapeutic targets and approaches aiming at the development of novel compounds against this parasite [3–5]. Here, we present data that validates *T*. *cruzi N-*myristoyltransferase as a potential chemotherapeutic target in mammal-dwelling stages of the parasite. *N-*myristoyltransferase has been intensively studied as a drug target for fungal and parasitic infections [41–44]. Nonetheless, despite the obvious importance of this lipid modification in *T*. *cruzi*, until recently no much effort has been taken in exploring TcNMT as a potential drug target in Chagas disease. A recent study by Roberts et al. [25], however, has performed the first molecular and biochemical characterization of *Tc*NMT. These authors showed that NMT is found in crude lysates of all stages of *T*. *cruzi*. When they tried to knockout the two copies of the *TcNMT*, they found that was only feasible if an ectopic copy of the gene was constitutively expressed by the parasite. These results clearly point out to the importance of the NMT for the survival of the noninfective epimastigote form of the parasite. In that study, the pyrazole sulfonamide NMT inhibitor **2** was tested with the recombinant enzyme and epimastigotes. Although that drug showed potent in vitro and in vivo inhibitory activity against *T*. *brucei*, its inhibitory activity in *T*. *cruzi* was considerably much lower. This compound was 13- to 23-fold less potent against recombinant *Tc*NMT than *Tb*NMT, and ~1,000-fold less potent against epimastigotes than was previously reported for *T*. *brucei* [25]. This discrepancy in drug potency against the enzymes might be caused by the dissimilarities in the amino acid composition surrounding the active site of the two NMT orthologs.

In the present study, the amino acid sequence analysis clearly showed conservation of residues predicted to be essential for NMT activity. Interestingly, two 30- and 22-mer insertions located at the N-terminus and C-terminus, respectively, are found in *Tc*NMT, suggesting additional secondary and tertiary structures that could affect *Tc*NMT activity relative to *Tb*NMT, *Lm*NMT, and human NMT (*Hs*NMT). The first insertion located near to N-terminus is also present but shorter in *Tb*NMT and *Lm*NMT; the second, although present and longer (28-mer) in *Tb*NMT, is clearly absent in *Lm*NMT. These differences between different protozoan and human NMTs could eventually be exploited in the development of specific NMT inhibitors. NMTs from *L*. *major* and *T*. *brucei* have been shown to partition between membrane and cytosolic fractions [14,24]. Here, we show by colocalization with BiP that *Tc*NMT is at least partially associated to the ER. Moreover, we provide evidence that NMT is constitutively expressed, and most importantly present in those stages (i.e., ICA and TCT) of *T*. *cruzi* that maintain the disease chronic in mammals, further highlighting the importance of this enzyme.

Screening of a 62,000 diversity-based compound library against *T*. *brucei* NMT, followed by a subsequent chemistry optimization program, led to the discovery of potent inhibitors that cured African trypanosomiasis *in vivo* [17]. In this study, we tested eight inhibitors against intracellular (ICA and TCT) forms *T*. *cruzi*. We presented evidence of strong inhibition of intracellular amastigote proliferation particularly with compounds **1**, **5**, and **8**. Importantly, they did not show cytotoxic effects against mammalian cells at effective trypanocidal concentrations. Interestingly, in our study compound **2** showed no trypanocidal activity against purified ICA. In contrast, treatment with compound **2** resulted in rapid killing of *T*. *brucei* trypanosomes both *in vivo* and *in vitro* [17]. For reasons that are not completely understood, *T*. *brucei* appears to be hypersensitive to NMT inhibition. Treatment with compound **2** in *T*. *brucei* results in the “BigEye” phenotype, where endocytosis is disturbed, leading to expansion of the flagellar pocket. As extensively discussed by Roberts et al. [25], the differences in susceptibility to NMT inhibition between *T*. *cruzi* and *T*. *brucei* could be due to differences in endocytosis or in the rate of plasma membrane turnover between these two species. Additionally, differences in cellular uptake and efflux of the inhibitors, as well as, differences in essential biological functions requiring *N-*myristoylation, could also play a role in the decreased potency of DDD compounds against *T*. *cruzi*.

In addition, we have demonstrated the “on target” effect of these compounds by metabolic labeling with myristic acid, azide. In-gel western blots of lysates from parasites showed a significant decrease in protein *N-*myristoylation relative to the untreated controls. Strikingly, Epi were less susceptible to these compounds, which might be explained by the *Tc*NMT overexpression observed in this stage of the parasite. This overexpression suggests a compensation mechanism for the inhibition of TcNMT, confirming the specificity of the inhibitors. Overall, it is likely that the effects of the tested compounds are a result of several downstream events, as this enzyme has over 100 putative substrates [21]. The lead compounds hold great potential to be explored as anti-Chagas disease agents.

## Supporting Information

S1 Fig Anti-LmNMT and anti-TbNMT recognize TcNMT. Immunofluorescence microscopy of Epi stained with anti-LmNMT and anti-TbNMT (red), co-stained with DAPI (blue) to reveal positions of the nucleus and kinetoplast (blue). Scale bar, 5 μm.
(TIF)

S2 Fig DDD compounds inhibit intracellular proliferation of *T*. *cruzi*. The multiparametric data obtained on a cell-by-cell basis by HCI was analyzed to determine several parameters associated to infection of host cells by *T*. *cruzi*. (**A** and **B**) Percentage of cells infected with at least one parasite (percentage of infected cells). (**C** and **D**) Percentage of cells infected with at least three parasites (percentage of cells in which the parasite proliferated). C, controls: BZ (800, 400, and 40 μM), Untreated, and DMSO.
(TIF)

S3 Fig DDD compound 2 is not trypanocidal against purified intracellular amastigotes. Total number of cells in each well was counted by HCI to evaluate the cytotoxicity of the compound **2** against purified ICA forms. C, controls: Untreated, DMSO, and H_2_O_2_.
(TIF)

S4 Fig DDD compounds are toxic against mammalian cells only at high concentrations. Total number of cells in each well was counted by HCI to evaluate the cytotoxicity of the compounds **1**, **5**, and **8** against U2OS cells. C, controls: Untreated, DMSO, and H_2_O_2_.
(TIF)

S5 Fig *T*. *cruzi* myristoylated proteins are not susceptible to base treatment. In-gel western blot of Epi lysate was treated (+ NaOH) or not (- NaOH) with 0.2 M NaOH in methanol to remove any base-labile hydroxy- or thioester-linked myristic acid azide, followed by “click” reaction with biotin alkyne. IRDye 800CW streptavidin was used to detect myristoylated proteins.
(TIF)

S6 Fig DDD compounds do not affect protein translation. Fluorographs of PVDF membranes of [^35^S]-methionine-labeled lysates from Epi, ICA, and TCT forms treated or not with 10 μM of the compound **1**, **5**, or **8**.
(TIF)
