# Supplementary figures and images for: Validation of N-myristoyltransferase as Potential Chemotherapeutic Target in Mammal-Dwelling Stages of Trypanosoma cruzi
Source: PLoS Negl Trop Dis. 2016 Apr 29;10(4):e0004540. doi: 10.1371/journal.pntd.0004540 (PMC4851402; doi:10.1371/journal.pntd.0004540)

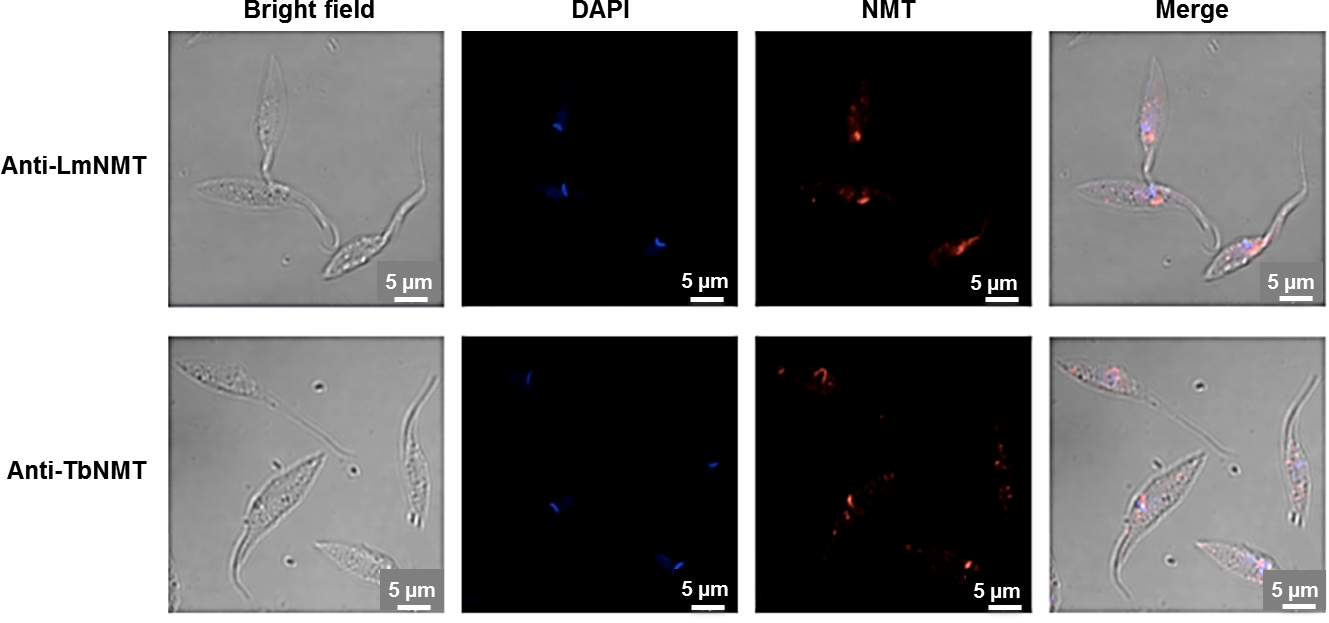

Supplement: S1 Fig — Immunofluorescence microscopy of Epi stained with anti-LmNMT and anti-TbNMT (red), co-stained with DAPI (blue) to reveal positions of the nucleus and kinetoplast (blue). Scale bar, 5 μm. (TIF) [file pntd.0004540.s001.tif]

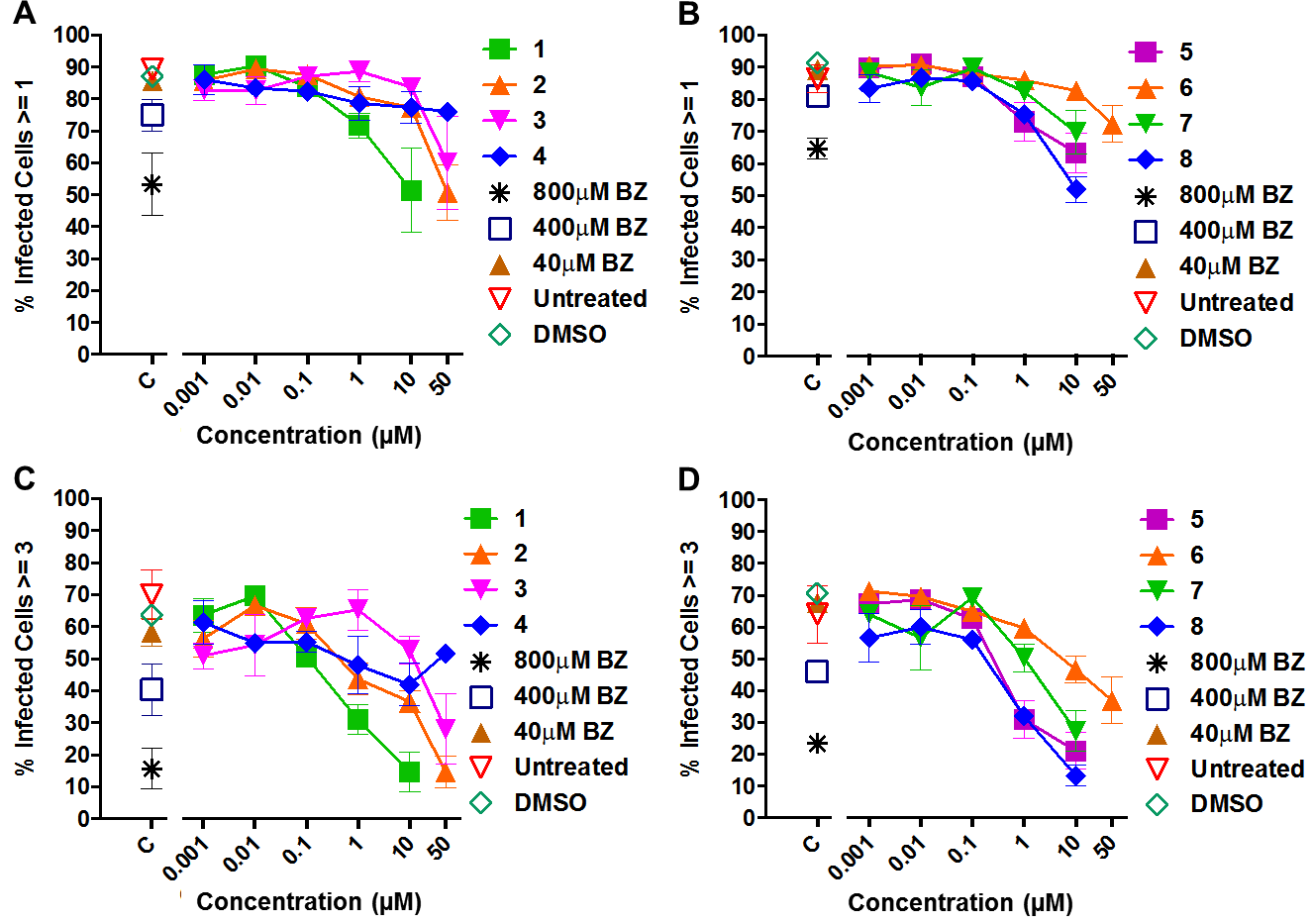

Supplement: S2 Fig — The multiparametric data obtained on a cell-by-cell basis by HCI was analyzed to determine several parameters associated to infection of host cells by T. cruzi. (A and B) Percentage of cells infected with at least one parasite (percentage of infected cells). (C and D) Percentage of cells infected with at least three parasites (percentage of cells in which the parasite proliferated). C, controls: BZ (800, 400, and 40 μM), Untreated, and DMSO. (TIF) [file pntd.0004540.s002.tif]

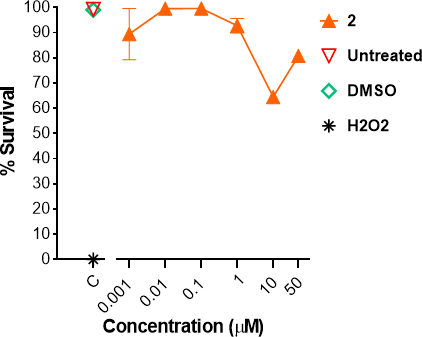

Supplement: S3 Fig — Total number of cells in each well was counted by HCI to evaluate the cytotoxicity of the compound 2 against purified ICA forms. C, controls: Untreated, DMSO, and H2O2. (TIF) [file pntd.0004540.s003.tif]

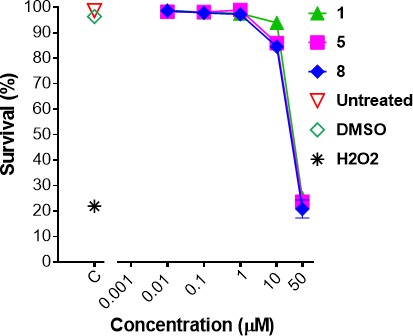

Supplement: S4 Fig — Total number of cells in each well was counted by HCI to evaluate the cytotoxicity of the compounds 1, 5, and 8 against U2OS cells. C, controls: Untreated, DMSO, and H2O2. (TIF) [file pntd.0004540.s004.tif]

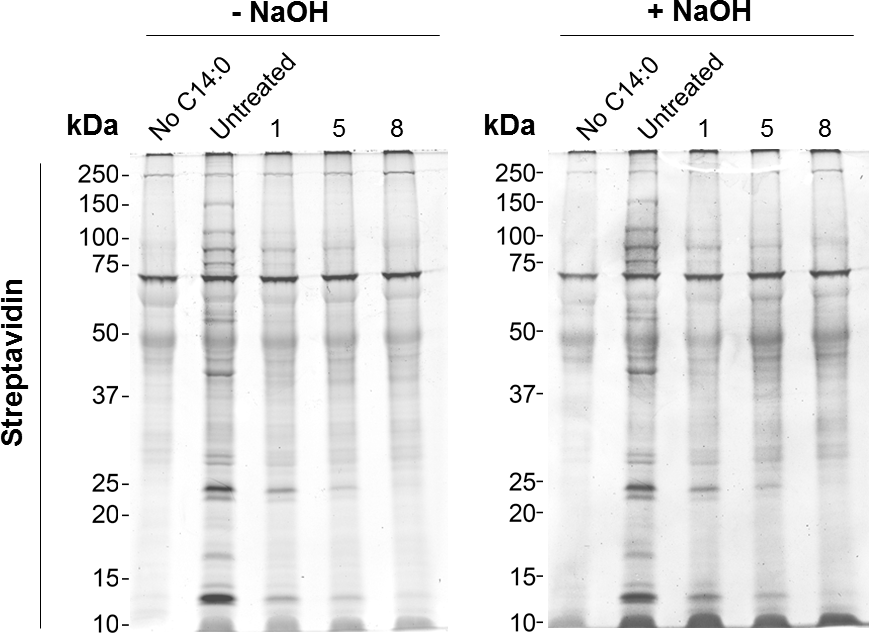

Supplement: S5 Fig — In-gel western blot of Epi lysate was treated (+ NaOH) or not (- NaOH) with 0.2 M NaOH in methanol to remove any base-labile hydroxy- or thioester-linked myristic acid azide, followed by “click” reaction with biotin alkyne. IRDye 800CW streptavidin was used to detect myristoylated proteins. (TIF) [file pntd.0004540.s005.tif]

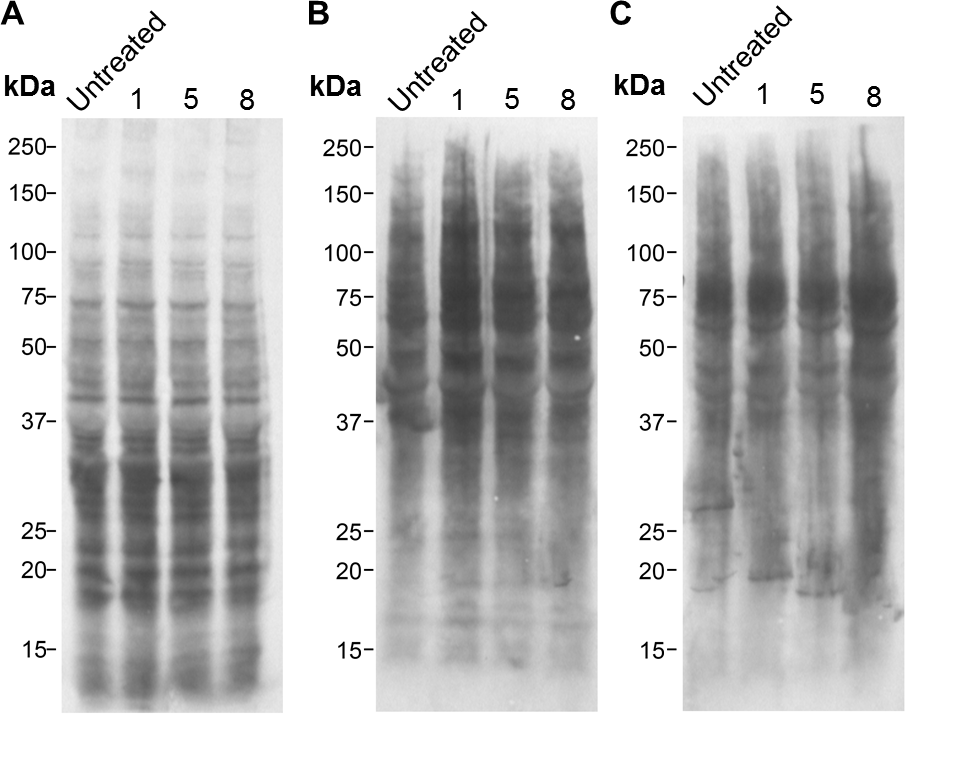

Supplement: S6 Fig — Fluorographs of PVDF membranes of [35S]-methionine-labeled lysates from Epi, ICA, and TCT forms treated or not with 10 μM of the compound 1, 5, or 8. (TIF) [file pntd.0004540.s006.tif]
